# Three cases with chronic obsessive compulsive disorder report gains in wellbeing and function following rituximab treatment

**DOI:** 10.1038/s41380-024-02750-y

**Published:** 2024-09-21

**Authors:** Maike Gallwitz, Isa Lindqvist, Jan Mulder, Annica J. Rasmusson, Anders Larsson, Evelina Husén, Jesper Borin, Peter J. van der Spek, Nour Sabbagh, Anna Widgren, Jonas Bergquist, Simon Cervenka, Joachim Burman, Janet L. Cunningham

**Affiliations:** 1 Department of Medical Sciences, Psychiatry, Uppsala University, Uppsala, Sweden; 2 Department of Neuroscience, Karolinska Institute, Stockholm, Sweden; 3 Department of Medical Sciences, Clinical Chemistry, Uppsala University, Uppsala, Sweden; 4 Department of Pathology and Clinical Bioinformatics, Erasmus MC, Rotterdam, The Netherlands; 5 Department of Chemistry – BMC, Analytical Chemistry and Neurochemistry, Uppsala University, Uppsala, Sweden; 6 Centre for Psychiatry Research, Department of Clinical Neuroscience, Karolinska Institute and Stockholm Health Care Services, Region Stockholm, Stockholm, Sweden; 7 Department of Medical Sciences, Translational Neurology, Uppsala University, Uppsala, Sweden

**Keywords:** Diagnostic markers, Psychology

## Abstract

Immunological aetiology is supported for a subgroup with obsessive compulsive disorder (OCD) and conceptualized as autoimmune OCD. The longitudinal clinical course is detailed for three severely ill cases with OCD and indications of immunological involvement with off-label rituximab treatment every six months. All cases showed clear and sustained gains regarding symptom burden and function for over 2.5 years. Brief Psychiatric Rating Scale and Yale-Brown Obsessive-Compulsive Inventory Scale scores decreased 67-100% and 44-92%, respectively. These complex cases, prior to rituximab, had very low functioning and disease duration has been eight, nine and 16 years respectively. All three patients had been unsuccessfully treated with at least two antidepressants or anxiolytics, one neuroleptic and cognitive behavioural therapy. Clinical phenotypes and findings were suggestive of possible autoimmune OCD. Indirect immunohistochemistry detected cerebral spinal fluid (CSF) antibodies in all three cases including a novel anti-neuronal staining pattern against mouse thalamic cells. Exploratory analyses of CSF markers and proteomics identified elevated levels of sCD27 and markers indicative of complement pathway activation when compared to CSF from healthy controls. Multidisciplinary collaboration, advanced clinical investigations and rituximab treatment are feasible in a psychiatric setting. The case histories provide a proof of principle for the newly proposed criteria for autoimmune OCD. The findings suggest that clinical red flags and biological measures may predict rituximab response in chronic treatment-resistant OCD. The report provides orientation that may inform the hypotheses and design of future treatment trials.

## Background

An increasing amount of data links OCD- and psychosis-like symptoms to mild forms of autoimmune encephalitis (AE) [1, 2]. AE was initially described with mainly neurological symptoms and associated with magnetic resonance imaging (MRI) signs of structural damage and brain edema, cerebrospinal fluid (CSF) pleocytosis, CSF-specific oligoclonal bands or elevated CSF IgG index, as well as elevated neuronal damage markers [3–5] and brain-reactive auto-antibodies (Ab) [6, 7]. More recently, obsessions, psychotic and affective symptoms and cognitive deficits have been linked to AE [8, 9]. New brain targets for autoimmunity associated with various psychiatric manifestations are emerging [2, 10–16]. Several links between OCD and autoimmunity are implied by for example the elevated OCD comorbidity with autoimmune disease [17–19]. Psychiatric symptoms have successfully been reduced with immunotherapy in recent observational studies [20, 21].

OCD patients suffer from strikingly low quality of life and severe loss of function [22, 23]. About fifty percent do not respond to standard treatment options [24]. Immunotherapy may be effective for OCD with immunologic aetiology, and optimal effect may rely on its timely initiation [25–27]. Many of the described cases do not have distinct radiological features or known anti-neuronal antibodies and there is a need to establish and validate new methods to reliably identify OCD patients possibly benefitting from such treatment [9]. Eleven clinical red flags have recently been proposed that should motivate further investigation to improve the detection of autoimmune OCD [1, 28–30] (see Table 1). Indirect support for antibody-mediated pathology may be provided by increased intrathecal immunoglobulin (Ig) G and oligoclonal bands (OCBs), or pathologic brain immunohistochemistry (IHC) findings [29]. A growing hypothesis is that the presence of red flags may predict response to immunotherapy, even when distinct MRI abnormalities and known autoantibodies in CSF or serum are absent [31, 32].

**Table 1:** Red flag symptoms for potential autoimmune OCD as suggested by Endres et al. [30], their presence in the presented cases. See footnotes for suggestions for revised phrasing.

Red Flag	Case 1	Case 2	Case 3	Comment
**1) (SUB)ACUTE ONSET OF OCD (** < **3 MONTHS)**^**a**^	**Yes**	**Yes**	**Maybe**	Case 3: accentuation of OCD
**2) TREATMENT RESISTANCE DESPITE GUIDELINE-BASED THERAPY**^**b**^	**Yes**	**Yes**	**Yes**	
**3) ATYPICAL AGE OF ONSET (EARLY CHILDHOOD OR LATER ADULTHOOD)**	No	No	No	
**4) ATYPICAL PRESENTATION OF OCD**	**Yes**	**Yes**	**Yes**	Cases 1-2: severe hypersomnia; Cases 1-3: loss of function due to disproportionate cognitive deficits
**5) ACCOMPANYING NEUROLOGICAL SIGNS: MOVEMENT DISORDER, FOCAL NEUROLOGICAL DEFICITS; NEW SEIZURES; NEW HEADACHE**	No	**Yes**	**Maybe**	Case 2: choreatic movements; Case 3: seizures
**6) AUTONOMIC DYSFUNCTION**	No	No	No	
**7) ADVERSE RESPONSE TO ANTIPSYCOITICS**	No	No	No	
**8) TEMPORAL ASSOCIATION OF ONSET WITH INFECTIONS**^**c, d**^	No	**Maybe**	**Maybe**	Case 2-3: Self-reported, unverified
**9) COMORBID AUTOIMMUNE DISEASE**	No	**Yes**	**Maybe**	Case 2: Kawasaki’s disease, Case 3: suspected APS
**10) COMORBID MALIGNANCIES (SUCH AS OVARIAN TERATOMA)**	No	No	No	
**11) SUSPICIOUS ALTERTIONS IN INVESTIGATIONS:**	**Yes**	**Yes**	**Yes**	See below
A) SERUM: AUTOANTIBODIES (NEURONAL, ANA)	No	Yes	No	Case 2: DRD-1 antibodies, (streptococcal abs. not tested)
B) EEG: SIGNS OF ENCEPHALOPATHY	-	No	No	
C) MRI: BASAL GANGLIA/MESIOTEMPORAL HYPERINTENSITIES, INFLAMMATORY LESIONS	No	No	Maybe	Case 3: punctiform subcortical white matter changes
D) 18FDG-PET: ENCEPHALITIC PATTERNS WITH DISTURBED METABOLISM IN BASAL-GANGLIA, AND ALONG CORTICAL OR TEMPORAL REGIONS	Yes	-	No	Case 1: low uptake in anterior cingulum, right cerebellar hemisphere and medial of the posterior horn
E) CSF: PLEOCYTOSIS, OCBs, NEURONAL AUTOANTIBODIES, HIGH IgG INDICIES, DAMAGE MARKERS	Yes	No	Yes	Case 1: increased IgG index; Cases 1 + 3: OCBs

*APS* antiphospholipid syndrome, *ANA* antinuclear antibodies, *DRD-1* Dopamine receptor 1, *CSF* cerebrospinal fluid, *OCBs* CSF-specific oligoclonal bands

Suggested revision (Comment):

^a^a (sub)acute (<3 months) onset or accentuation of OCD.

^b^Add: Long-term (>2 years) inability to follow guidelines because of side-effects.

^c^The criterion is subjective and could potentially be improved by objective measures.

^d^Temporal association of onset or clear exacerbation with infections, allergies or exacerbation of underlying immune disorder, and/or temporal association of amelioration with immune-modulatory treatment. (The criterion is hard to prove and distinguish from an undulating course of OCD).

This study provides longitudinal clinical data characterizing three severely ill, previously treatment-resistant cases with signs of autoimmune OCD where rituximab treatment was followed by clear gains regarding symptom burden and function. Exploratory analyses of inflammatory markers, proteomics and neuronal antibodies were then performed to identify markers that should be evaluated for value in predicting response to immunotherapy in future treatment trials.

## Material and methods

### Statement of ethics

The research was conducted in accordance with the World Medical Association Declaration of Helsinki. Ethical approval was acquired by the Regional Ethical Review Board in Uppsala, Sweden; Dnr 2012/081 and 2014/148. Written informed consent was obtained from all participants. All three cases have submitted written approval of their respective case presentations.

### Cases

Cases were referred to the Immunopsychiatry Clinic at Uppsala University Hospital [33, 34]. Their long-standing psychiatric symptoms were not responsive to standard treatments or patients reported intolerable side effects. Treatment decision was based on a multidisciplinary evaluation of signs of immunological involvement (comorbid autoimmune disease and/or serum autoantibodies, CSF-specific oligoclonal bands, elevated CSF IgG-index and in combination with this white matter changes on MRI and/or not-normal brain FDG-PET (for details see Table 1)), and potential risks and benefits were assessed in accordance with The Helsinki declaration. Exact age is not presented to preserve anonymity. Two cases are male, one female.

### Controls

Control plasma (*n* = 51) and CSF (*n* = 6) was obtained from university employees and students without a history of psychiatric illness based on a semi-structured psychiatric diagnostic interview, clinical health examination and questionnaires. Controls had a mean age of 22 years (range 18–46); the male:female ratio was 13:38. Controls where CSF was available had a mean age of 29 years (range 27–33); the male:female ratio was 1:2.

### Psychiatric and neurological assessment

Assessment of cases involved psychiatric interview, neurological examination and a review of medical records. Obsessions and compulsions were characterized using the Yale-Brown Obsessive-Compulsive Inventory Scale (Y-BOCS) [35]. A relapsing-remitting course of illness was defined as major fluctuations of symptoms not related to medication. The 24-item Brief Psychiatric Rating Scale-Expanded (BPRS-E) [36] was administered at every clinical evaluation and in conjunction with rituximab infusions. Daily life function was assessed using Sheehan Disability Scale (SDS) [37], and by inquiring about ability to carry out activities of daily living (ADL) and to work or attend school. MRI, positron emission tomography (PET) and EEG data were gathered where available. The presence of Red Flag criteria for autoimmune OCD as suggested by Endres et al. [30]. was assessed retrospectively. See Supplementary Methods for complete list of clinical investigations.

### Rituximab treatment

After pre-medication with 1 g paracetamol p.o., 10 mg cetirizine p.o. and 125 mg methylprednisolone i.v., 1000 mg rituximab in 500 ml 0.9% sodium chloride was administered as i.v. infusion. Patients were monitored for adverse reactions during the procedure. Treatment was repeated approximately every 6 months over 2.5 to 3 years. The effect of rituximab on the B cell lineage was assessed after a month by flow cytometry targeting CD19 and CD20.

### Blood samples and CSF collection and analyses

Serum, plasma and CSF collected at repeated timepoints before and after initiation of rituximab treatment was stored at -80°C. Inflammatory markers were measured using a commercially available assay according to the manufactures protocol (MULTI-ARRAY Assay System, Meso Scale Discovery; see Supplementary Table 2 and Supplementary Methods). Proteomics analysis of baseline CSF from cases and controls with label free mass spectrometry, is described in the Supplementary Methods section. The sample preparation and mass spectrometry, including data handling, followed the workflow described previously [38].

### Indirect immunohistochemistry

Brains were collected from 4% paraformaldehyde-perfused p56 mice, cut to 16 µm slices and attached to superfrost plus slides. Slides were incubated overnight with 1:4-diluted CSF from cases prior to rituximab treatment or controls, respectively. Thereafter, secondary horseradish peroxidase-conjugated anti-human IgG, IgA or IgM was added and labelled separately with tyramide signal amplification fluorescent cyanine-dyes. A blinded digital analysis provided the mean staining intensity in different brain areas for cases vs controls. IgG staining-patterns were then qualitatively assessed in whole sagittal mouse brain tissue sections by a blinded evaluator. Patterns were categorized as synaptic-like, neuronal-like, cytosolic or purkinje-cells based on comparisons with well-characterized proteins [39] (see Supplementary Methods).

## Results

### Clinical features and treatment effects

**Fig. 1:**
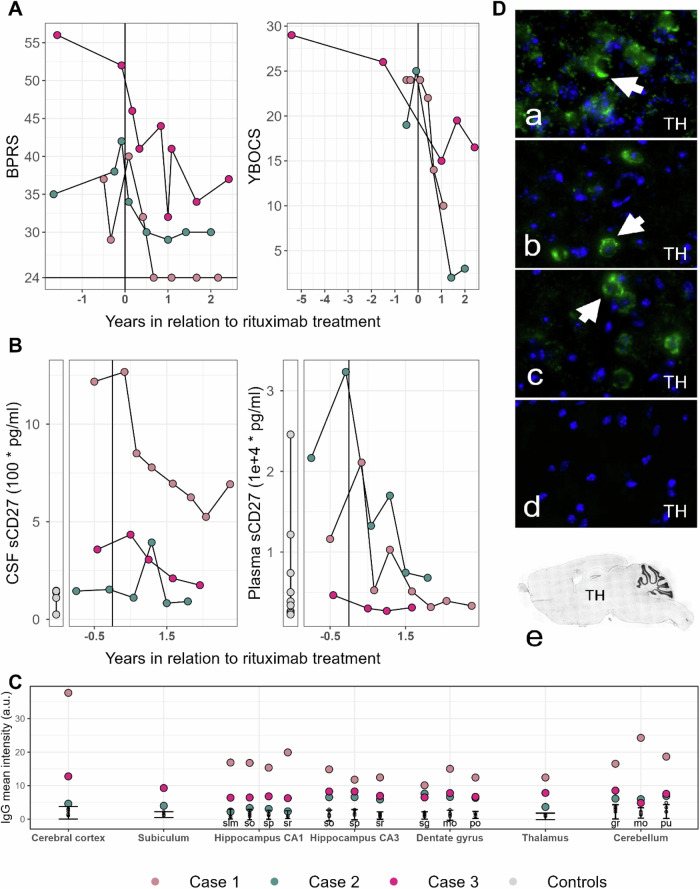
Longitudinal evaluation of symptoms in relation to rituximab treatment. **A** Longitudinal evaluation of symptoms was conducted with the symptom rating scales: Brief Psychiatric Rating Scale (BPRS) and Yale-Brown Obsessive-Compulsive Scale (YBOCS). The horizontal line indicates a BPRS score of 24 reflecting no current symptoms. **B** CSF and blood was repeatedly sampled at six-month intervals. Levels of sCD27 in CSF and plasma are shown compared to healthy controls, shown as individual grey dots (CSF *n* = 6, plasma *n* = 51). **C** Indirect IHC staining intensity for anti-mouse brain IgG counterstained with DAPI, using CSF. Shown are case and control mean signal intensities for seven brain regions corrected for background. Healthy controls (*n* = 6) shown as individual grey dots with an error bar ± 2 SD from mean control intensity. Subiculum was not available for Case 1. Subregions are indicated as follows: slm-lacunosum, so- oriens layer, sp- pyramidal layer, sr-radiatum layer, sg-granule cell layer, mo-molecular layer and po-polymorph layer, gr- granular layer and pu-purkinje layer. **D** Indirect IHC demonstrating CSF IgG reactivity (green) against neuronal structures in mouse thalamic regions with DAPI counterstaining (blue). Arrows indicate CSF autoantibody staining of a subset of cytosolic neuronal-like cells in the mouse thalamic region for all three cases (a-c) but not controls (d). Synaptic features around the cells are seen in case 1 (a) and 3 (c). Sagittal overview demonstrating location of thalamic regions (e).

All three presented cases displayed clinical and immunological abnormalities and matched three or more of the suggested Red Flags for potential autoimmune OCD (Table 1). Patients were systematically evaluated every 6 months for 2.5 years. Rituximab treatment was followed by clear improvement in general psychiatric and OCD-specific symptoms as measured by BPRS-E and Y-BOCS (Fig. 1A), and function (Tables 2, 3, and Supplementary Table 1).

**Table 2:** Case 1-3: Clinical characterization, heredity and immunological comorbidity.

	Case 1	Case 2	Case 3
**SEX**	Male	Male	Female
**AGE AT DEBUT**	Early adulthood	Pre-pubertal	Early adulthood
**HEREDITY**	OCD, anxiety	ADHD, autism, MS, skin infections	RA, IBD, thromboembolism, (endometriosis)^a^
**OCD DURATION**	Eight years	Nine years	~16 years
**COURSE OF OCD**	Recurrent-remittent with tics	Progressive with intermittent improvements, with tics	Progressive with intermittent improvements
**COMORBIDITY OF PROBABLE IMMUNOLOGICAL RELEVANCE**^b^	None known	Meningitis (age four), Kawasaki’s disease, urticaria, allergies, asthma. PANDAS and Kleine-Levin syndrome	Heterozygous for Factor V Leiden. Suspected APS, suspected endometriosis
**AFFECTIVE SYMPTOMS**	Anxiety, depression	Anxiety, depression, aggressive outbursts.	GAD, social phobia, periods of elated and depressed mood, emotional outbursts.
**SLEEP**	Irregular with hypersomnia	Irregular with hypersomnia and sleep attacks.	Irregular
**SENSORY**	Light- and sound sensitivity Hyperawareness of sensory input including touch	Light- and sound sensitivity.	Paraesthesia, light- and sound sensitivity, numbness. Skin twitching, muscle cramps
**MOTOR SYMPTOMS**		Walking in patterns, choreatic movements, “childish” behavior	
**COGNITIVE SYMPTOMS**	Reports reduced executive function, difficulty shifting focus and “getting stuck” when executing automatic behaviors. Observable obsessive rumination.	Reports reduced executive function, difficulties with shifting focus, logic and time perception. Observable perseveration and verbigeration.	Reports reduced executive function, difficulty shifting focus, dizziness, fatigue, brain fog, subjective aphasia and headaches. Observable difficulties in organising thoughts.
**OTHERS**		Extreme weight gain	Reports swollen joints, dyspnea

*ADHD* Attention deficit/hyperactivity disorder, *MS* Multiple Sclerosis, *RA* Rheumatoid Arthritis, *IBD* inflammatory bowel disease, *PANDAS* pediatric autoimmune neuropsychiatric disorders associated with streptococcal infections, *APS* antiphospholipid syndrome, *GAD* general anxiety disorder.

^a^Endometriosis has not yet been classified as an autoimmune condition, but may increase the risk for autoimmune conditions.

^b^Cases were all tested negative for pleocytosis, albumin and albumin quotient levels, anti-nuclear antibodies including NMDAr-Abs (Fixed cell assay, Euroimmune), antinuclear antibodies, borrelia serology.

**Table 3:** Case 1-3: Immunological and radiological findings, presence of Red Flags, and changes 24 months after Rituximab treatment.

	Case 1	Case 2	Case 3
**FINDINGS**	Elevated CSF-lgG, Unmatched OCBs; CaM II kinase activity (150%); EEG: Not done; MRI: Normal; 18FDG-PET: Low uptake in anterior cingulum, right cerebellar hemisphere and medial of the posterior horn	Positive DRD1-Ab and beta-Tub Ab, EEG Normal, MRI: Normal. 18FDG-PET: Not done	Unmatched OCBs, CaM II kinase activity (150%), EEG Normal. MRI with Hyperintense punctiform subcortical white substance changes, 18FDG-PET: Normal
**RED FLAGS AUTOIMMUNE OCD**	4 definite	6 definite + 1 suspected	3 definite + 4 suspected
**RITUXIMAB TREATMENT**	Six rounds a 1 g every 6 months, starting in late 20 s	Five rounds a 1 g every 6 months, starting in early adulthood	Five rounds a 1 g every 6 months, starting in mid-30s
**Change 24 months after rituximab initiation**			
**AFFECTIVE SYMPTOMS**	Improved for 2 years then relapse (after 2.5 years)	Improved	Improved
**SLEEP**	Normalized (from 12 h/d to 7,5 h/d)	Normalized without sleep medication (from 12 h/d to 8-9 h/d)	Improved without sleep medication, improved energy
**MOTOR SYMPTOMS**	-	Improved	-
**ABILITY TO WORK/ SCHOOL**	Increased from 0% to 100%	Improved	Increased from O to 50%
**ADL FUNCTIONS**	Improved. Completed full time studies and an apprenticeship	Improved. Is more independent in cooking and cleaning. Started part-time distance studies	Improved. Started new part-time work
**COGNITION**	Reports improved executive function, ability to shift focus, increased initiative and interest. Reports no longer “getting stuck”. Observably less rumination.	Reports improved executive function, ability to shift focus and read advanced text. Observably less perseveration and verbigeration and improved formal communication.	Reports improved executive function, ability to shift focus and remission of subjective aphasia. Observably improved ability to organise thoughts and formal communication. During 3 years, no periods of elevated mood were observed and good adherence to treatment.
**MEDICATION**	Risperidone discontinued; Sertraline was reduced from 200 to 50 mg	Discontinued all psychiatric medications (SSRI, anxiolytics, CS)	No medication

*OCBs* Oligoclonal bands, *CSF* Cerebral Spinal Fluid, *DRD1* dopamine receptor 1, *SSRI* Selective serotonin reuptake inhibitors, *CS* Corticosteroid.

#### Case 1

Male with long-standing recurrent-remitting OCD, disturbed sleep and pronounced loss of function.

In his early twenties, case 1 suddenly developed recurrent-remittent OCD, insomnia/hypersomnia (up to 36 h of sleep) and depressive symptoms. He was ruminating and worrying about “everything”, describing his brain as “on fire”. He experienced previously automated complex motor activities such as opening a door as subdivided into a series of conscious decisions (first taking the handle, then noting its’ temperature and so on). where he could “lose his way” and have to restart the sequence from the beginning. Difficulties with decision making and being “stuck” in activities debilitated his social life, family relations and autonomy. For several years he was dependent on his parents, intermitted with some months where he could work.

The patient was referred to our clinic after eight years of unsuccessful traditional psychological and pharmacological therapy against OCD and recurring pronounced loss of function. ^18^Fludeoxyglucose (FDG)-PET scan showed low metabolic activity in the anterior cingulum and right cerebellum, and CSF analysis indicated intrathecal antibody production with OCBs and elevated IgG-index. He was treated with six rounds of 1000 mg rituximab at six-monthly intervals. Over the first two treatment years, he reported “feeling extremely well” and his capacity to attend school increased from 0 to 100%, together with normalization of intrathecal antibody production.

However, some months after his final treatment and nearly two and a half years without symptoms, within days of starting a new employment, he relapsed into nightly rumination, difficulties making decisions and obsessions. Flow cytometry at that time indicated sustained rituximab effect on the B cell lineage with very suppressed levels of CD20-positive B cells (0,1%). Several explanations for the relapse are possible. The improvements may have been solely or partially a placebo effect and the relapse could hence reflect the natural relapsing-remitting course of OCD symptoms. Complete symptom remission and high function for several years are however unprecedented in this patient´s OCD history. Starting this new job was linked with perceived high expectations from his surroundings, which may have triggered fear and stress. The relapse occurred a couple of months after discontinuation of sertraline, which may have augmented his vulnerability, and coincided with the first days at a new job. In support of the latter, reintroduction of antidepressant treatment improved the symptoms.

#### Case 2

Male with progressive severe OCD, tics, disturbed sleep and aggressive outbursts since age ten.

Case 2 is a male with co-morbid learning disorder, heredity for attention deficit and hyperactivity disorder (ADHD) and autism, and a history of recurring impetigo. He was afflicted by sudden-onset OCD and tics at age ten. He also deteriorated regarding autonomy, social and intellectual skills and with marked hypersomnia, hyperphagia and hypersexuality and was diagnosed with Kleine-Levin syndrome [40]. Exacerbations of his OCD and tics coincided with recurrent streptococcus infections that continued into adulthood. Symptoms did not respond to CBT, antidepressants, neuroleptics or mood stabilizers, but temporarily improved with antibiotic and intravenous immunoglobulin (IVIG) treatment. These observations led to the diagnosis of PANDAS. He was referred to our clinic as a young adult, relatively stabilized. A few months later, after prophylactic antibiotic was removed, he was successfully treated for sepsis, secondary to a surgical procedure but relapsed with loss of function, repetitive speech and behaviours, sleeping disorder and obsessions.

Standard immunological investigation showed no abnormalities (normal IgG subclasses, complement components and B and T cell function, no evidence for human immunodeficiency virus, tuberculosis or other ongoing infection). Grounds to suspect autoimmune OCD were provided by repeated post-infectious deteriorations.

The patient was treated with five rounds of 1000 mg rituximab. Subsequently, his sleep pattern normalized, he started to take part in household chores and to conduct part-time studies from home, still somewhat affected by difficulties concentrating. He discontinued all psychiatric medications. Since completion of the rituximab treatment, he remains unmedicated and reports a sustained feeling of being mentally “better than ever” that has been sustained for over three years. Part of these gains may be ascribed spontaneous remission which has been described for Kleine-Levin syndrome [41, 42]. However, symptoms gradually improved from about four months; a time course resembling that of clinical and radiological improvement after rituximab treatment in e.g. MS, [43, 44].

#### Case 3

Female with emotional outbursts, neurologic symptoms and bodily obsessions since early adulthood.

Case 3 displayed subclinical fearfulness since early childhood. She has heredity for rheumatoid arthritis (RA), inflammatory bowel disease (IBD) and endometriosis and suffered from dysmenorrhea from menarche. From her teens she worried about physical appearance and symmetry. Grades dropped during the final school years, but she was able to proceed to academic studies. Coinciding with a tick bite in her twenties where Borrelia infection was suspected but not verified, she experienced worsened sleeping problems, health and symmetry obsessions and difficulties concentrating. Abdominal pain and dysmenorrhea was also aggravated. At referral to general psychiatry, she initially presented with elated mood, and was diagnosed with OCD, generalized anxiety disorder (GAD) and social phobia. Recurrent episodes of elevated and depressed mood were later interpreted as bipolar disorder and treatment was for years mainly tailored to this. Over a decade, various psychiatric medications were tried without full effect, sometimes tolerated for only days because of side effects or fear of side effects. OCD symptoms such as obsessiveness about medication, side effects and bodily symptoms, may have contributed to suboptimal treatment.

At referral to our clinic, the woman’s communication and thoughts were markedly disorganised, with difficulties recapitulating her medical history and relating it to a timeline. She presented subtle neurological symptoms and hadn´t been able to work for four years. Immunological assessment revealed CSF-specific oligoclonal IgG bands, MRI showed punctiform subcortical white matter changes, and this together with heredity for autoimmune diseases and the atypical clinical presentation provided grounds to suspect autoimmune OCD. Levels of autoantibodies consistent with antiphospholipid syndrome were also modestly elevated but no clinical manifestations (e.g. miscarriages, thrombosis) supported the diagnosis.

The patient received five rounds of 1000 mg rituximab every 6 months. During this period, she also twice received antibiotics for tonsillitis. Despite previous difficulties, she had no problems complying with the treatment or assessments. Over the treatment period, the woman reported improved sleep, energy, cognitive and social functions including verbal capacity and reduced obsessiveness, and her communication became more organized. She was able to work part-time. No periods of mood shift were observed. However, a high level of anxiety persisted. She described episodes of neurological symptoms (numbness, skin twitching, focal muscle tension, paraesthesia) often in conjunction with wakening. These symptoms recessed for some days after the administration of 125 mg methylprednisolone given with rituximab and after a three-day high-dosage (1 g daily) i.v. hydrocortisone treatment, and became less frequent over the treatment years. It is unclear whether this somatic symptom cluster represents a separate pathology in this patient.

After the final treatment, the patient retained an intense fear that her pre-rituximab symptoms would return.

## Results from the biological studies

### Indirect Immunohistochemistry (IHC)

In IHC against mouse brain, CSF from all three cases yielded IgG staining intensity more than two standard deviations (+2SDs) above mean of controls for multiple brain regions (see Fig. 1C). Case 3 additionally displayed IgA and IgM staining intensity +2SDs against mouse hippocampus, whereas Case 1 and 2 IgA and IgM staining intensity was similar to controls (data not shown). Mean staining intensities were highest for Cases 1 and 3 in all investigated brain regions.

Manual examination of IgG staining patterns in whole sagittal mouse brain tissue sections identified synaptic-like structures throughout the sections for all the cases in differing proportions in conjunction with neuronal-like patterns. All three cases showed similar neuronal cytosolic IHC staining patterns in thalamus (see Fig. 1D). CSF from healthy controls displayed none of the patterns in any of the investigated anti-brain Ig-subclasses.

### Exploratory analysis of immunological markers

The immunological markers that in all three cases exceeded two standard deviations from control values form control values were the soluble form of the CD27 (sCD27), and C-C motif chemokine ligand 4 (CCL4) (see Supplementary Figs. 1 and 2). sCD27 levels were higher in CSF from case 1 and 3 before treatment initiation than for controls (*n* = 6), and were in both cases reduced in repeated samples after. Case 2 shows a similar pattern but sCD27 was pre-treatment more markedly elevated in plasma than in CSF (Fig. 1B and Supplementary Fig. 2). CCL4 levels were higher than in controls for all three cases pre-treatment and remained elevated. Moreover, levels of TNF superfamily member 13b (TNFSF13B) increased in all cases´ plasma over the treatment course (Supplementary Fig. 2).

### Proteomics

**Fig. 2:**
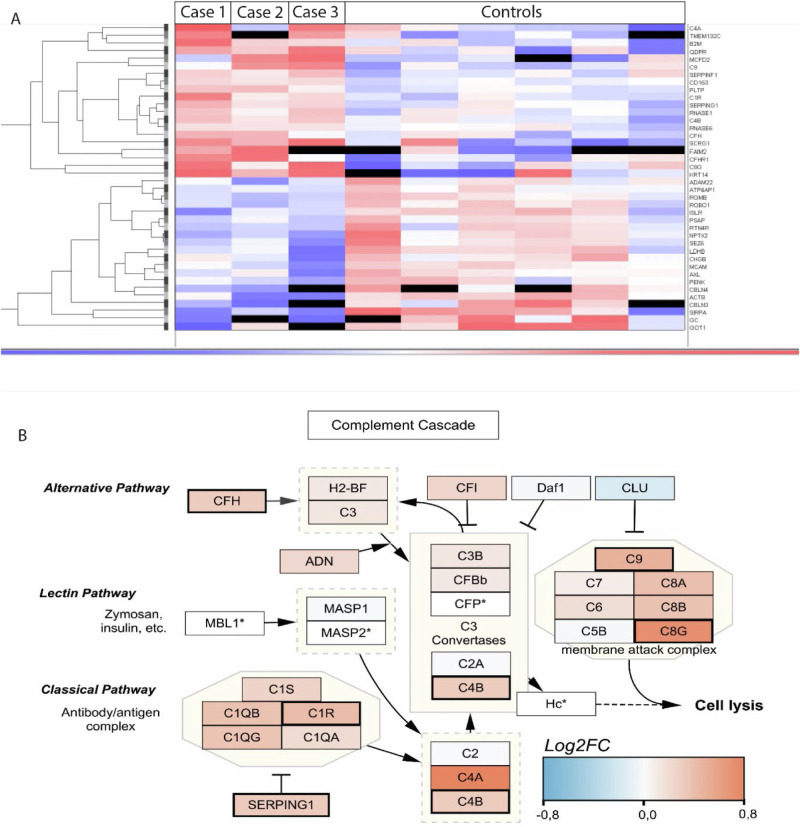
Heatmap visualization of the top 40 differentially expressed proteins in CSF when assessed with mass spectrometry. **A** A Heatmap visualization of the top 40 differentially expressed proteins in CSF when assessed with mass spectrometry. Proteins that were upregulated in the 3 independent OCD cases relative to controls are shown in red and the relative down regulation compared to the geometric mean after Log2 transformation is shown in blue. **B** Pathway analysis identified the the complement system. The protein interaction network is shown where nodes are coloured according to effect size of differential expression in cases versus controls. The increased border thickness of SERPING1, C1R, C4B, CFH, C8G and C9 indicates *p*-value < 0.05. *Proteins without representation were removed from the pathway or marked with an asterisk in cases of particular importance to pathway function.

Label free mass spectrometry was used as an unbiased approach to exploratory proteomics on CSF from the three cases relative to six healthy controls. A t-test was run to compare differential protein expression between OCD and control CSF. Two different methods of pathway enrichment from differential expression data both ranked cascades of the complement system on top (Supplementary Table 3). The complement pathway was upregulated in the three independent OCD cases shown in red vs relative down regulation shown in blue in the heatmap (Fig. 2A). The activation of the complement system has been associated with several autoimmune diseases where it promotes tissue injury and links to disease severity [45]. Notably, the most highly differentially expressed protein in the complement pathway measured by effect size, C4A, which reported a log2FC value of 0.83 and a *p*-value of 0.07, was high in Case 1 and 3 but did not reach significance as it was not elevated in Case2. Studies imply that the C4 isotypes can shape the immune response where C4a serve a crucial role in clearance of autoreactive B cells [46, 47]. The accompanying network illustrates the known biological interactions between differentially regulated complement components. Especially the classical and terminal pathways are standing out, both with regards to effect size and statistical significance (see Fig. 2B).

## Discussion

All three presented cases were previously treatment-resistant, fulfil the suggested criteria for possible Autoimmune OCD [29, 30] and show clear gains in function after repeated rituximab treatment. Notably, MRI and EEG were normal or showed unspecific CNS pathology. Extensive investigation provided support for immunological mechanisms including novel candidate inflammatory markers further discussed below. The longitudinal treatment response is in line with a successfully addressed underlying immunological pathology. Furthermore, these cases suggest that benefit from immunotherapy, such as rituximab, is possible even after prolonged periods of illness. The methodology applied for these cases could serve as a model for future studies with higher numbers of probands.

Rituximab is a very potent B-cell depleting agent, with doses as low as 25 mg being reported to lead to complete B cell depletion in peripheral blood [48]. Different dosing and intervals have been employed for treatment of human disease. 500–1000 mg every six months have been common in inflammatory neurological diseases [49, 50] and considerably higher doses are standard in oncological settings. After rituximab treatment B cell counts recover with variable speed. The new B cells are generally of a naïve phenotype, and it is not known to which extent they participate in the pathophysiology of disease [51]. Notably, as seen in patients with multiple sclerosis treated with rituximab the clinical improvement is gradual starting many months after treatment initiation and improvements can be noted over the course of several years [51]. A single dose of rituximab depletes the majority of B cell in peripheral blood, but B cells remain in secondary lymphoid organs although the cytokine landscape may be altered [52]. Unsurprisingly, studies find modulation in both humoral and T cell subsets in response to rituximab treatment [53]. More specifically significant decrease the T follicular helper cells as well as the regulatory T cell responses in both RA and MS [54, 55]. At this point, it is also not clear which dose and which dosing interval is optimal for any inflammatory disease. What constitutes effective dosing needs to be established on a disease basis as higher doses are associated with increased risk for adverse events. One recent small trial [56], where only one dose of rituximab was administered showed response in only 2/10 cases of treatment resistant OCD. However, the cases were neither pre-selected for red flags nor other immunological findings.

We do not believe that rituximab is effective for all cases with OCD and have chosen to be very conservative with off-label treatments. Two additional patients with symptoms of OCD have been treated. One patient, with comorbid thyroiditis, inflammatory bowel disease and recurrent vein thrombosis showed excellent response and complete remission but was found to have a different staining pattern and a family history suggesting specific genetic mechanisms that we are now investigating with the intent of reporting this case separately. Another case with OCD, still under investigation, presented with post-infectious regression, avoidant/restrictive food intake disorder, OCD and severe dystonia. This patient has shown partial response to cortisone and IVIG. One dose of rituximab was given but no clear benefits were seen and it was discontinued.

Clinically and functionally, the cases reported here are characterized by cognitive difficulties, substantial loss of executive functions in conjunction with disease debut and episodes, and a high symptom load and suffering leading to decade-long intense psychiatric care-seeking. Cases showed at least three of the previously described red flags [30] (Table 1). After rituximab treatment, care seeking behaviour almost completely ceased, symptoms subjectively improved and the patients objectively achieved higher function and better psychiatric health than before. Assessment of daily life function and cognitive testing suggestively should contribute important data in future cost-benefit considerations, and to clinically monitor and characterize disease burden and treatment effects for this patient subgroup.

Screening for antibodies with brain tissue-based assays has been suggested to provide valuable support for the diagnosis of autoimmune OCD [22, 57]. Here, indirect IHC using CSF on mouse brain yielded a distinct IgG staining intensity and localization for the three cases compared to CSF from controls, suggestive of brain-reactive autoantibodies. Whether or not these antibodies have direct pathogenic effects could not be assessed in the scope of this study. All cases displayed a similar thalamic staining pattern with neuronal cytosolic staining. Both neuronal and cilia staining have been reported in previous studies investigating autoimmune psychiatric disorders [58, 59]. The three OCD cases also commonly showed distinct cortical staining pattern which is open to further investigation in the context of a wider group of OCD patients with similar clinical phenotypes. Additional signs of elevated autoantibodies in the cases before treatment include CSF-specific OCBs and elevated IgG index, that normalized in case 1 and 3 as OCD symptoms recessed. Novel antibodies against mouse brain structures may be more specifically characterized in the future.

Case 2 had a history of repeated streptococcal infections and related autoimmunity (Kawasaki’s disease). An association between sudden onset of OCD-symptoms and tics with beta-hemolytic streptococcal (GAS) infections has been described, and has in children been termed Pediatric Autoimmune Neuropsychiatric Disorders Associated with Streptococcus (PANDAS) [39]. Several studies demonstrate autoantibodies against the basal ganglia and thalamus in serum and CSF of OCD patients and in children with PANDAS [60]. This patient displayed a staining pattern in CSF that targeted several brain regions but most distinctively parvalbumin-expressing interneurons in the oriens layer of the hippocampus and granular cells of the dentate gyrus. The pattern had greater IHC intensity in serum staining (data not shown). Notably, an interneuronal staining pattern has been described in other similar cases [60].

We identified inflammatory markers, sCD27 and CCL4, as candidates for further investigation. sCD27 is elevated in a wide range of neuroinflammatory states and autoimmune diseases [61–68]. sCD27 dynamics in the cases may reflect central vs peripheral inflammatory states that responded to treatment. The range of sCD27 CSF and plasma concentrations has recently been described in a large surgical population in relation to age, BMI and other clinical variables [69]. CCL4 plasma levels were consistently elevated in all cases, and CCL4 elevation has also been described in first episode psychosis [70], and in young adults with high levels of depressive symptoms [71]. Moreover, TNFSF13B plasma levels increased in all cases over the treatment course, which parallels observations after Rituximab treatment of other autoimmune diseases [72, 73].

In the exploratory proteomics, proteins related to the complement pathways were overrepresented in CSF from cases. The classical and alternative complement pathways as well as the terminal pathway appeared to be activated. This pattern can be seen in autoimmune diseases where antibodies (IgM and some IgG subclasses) activate the classical pathway [74, 75]. Elevated C4A in CSF, a marker for disease activity in multiple sclerosis [76], was noted for two of the three cases, notably the same two that showed elevated sCD27 in CSF. C4A has recently been shown to be elevated in some cases of first episode schizophrenia [77]. The elimination of synapses via synaptic pruning has furthermore been shown for other members of the complement cascade, namely C3 and the C1 complex [78, 79]. Despite the small sample size of this exploratory study, the effect size warrants further investigation of a possible causal relationship between complement activity, clinical progression and implications for predicting the treatment response. The approach we propose should not solely rely on the basic definition of clinical entities according to DSM/ICD. Rather extend more elaborated and specific patterns of phenotypes in combination with findings from additional investigations to define the entity of interest and motivate new treatment approaches. Future studies should, apart from higher case numbers, preferably include larger CSF control groups—however difficult to obtain—to allow for robust statistics and interpretation of results.

Taken together, three individuals severely ill with previously treatment-resistant and probable autoimmune OCD were treated with five or six courses of rituximab. Symptoms and function improved markedly, and this was sustained for years. Clinical red flags together with antineuronal antibodies, sCD27, C4A in CSF as well as peripheral levels of CCL4 emerged as candidate disease markers. The temporal association, longitudinal stability of clinical improvements and several immunological findings support a direct rituximab treatment effect, however inherently this case report is limited by the uncontrolled design and small sample size and cannot exclude unspecific effects such as placebo and regression–to-the-mean.

A necessary next step to sharpen the putative diagnostic tools and verify treatment response of suspected autoimmune OCD are trial with single case experimental designs or randomized controlled trials with repeated CSF sampling and extensive exploratory aims to better biologically differentiate responders from non-responders, which will contribute to the building of rational and tailored algorithms for the selection of patients and use of immunotherapy in OCD.

## Supplementary information

Supplementary Figure1

Supplementary Figure 2

Legends for Supplementary figures

Supplementary tables 1-3

## Data Availability

The data that support the findings of this study is available on request from the corresponding author (JLC, Uppsala University). The data is not publicly available due to privacy and ethical restrictions.
