# Supplementary material for: Three cases with chronic obsessive compulsive disorder report gains in wellbeing and function following rituximab treatment
Source: Mol Psychiatry. 2024 Sep 21;30(4):1396–406. doi: 10.1038/s41380-024-02750-y (PMC11919689; doi:10.1038/s41380-024-02750-y)

Plasma inflammatory marker levels before and after rituximab treatment compared to controls

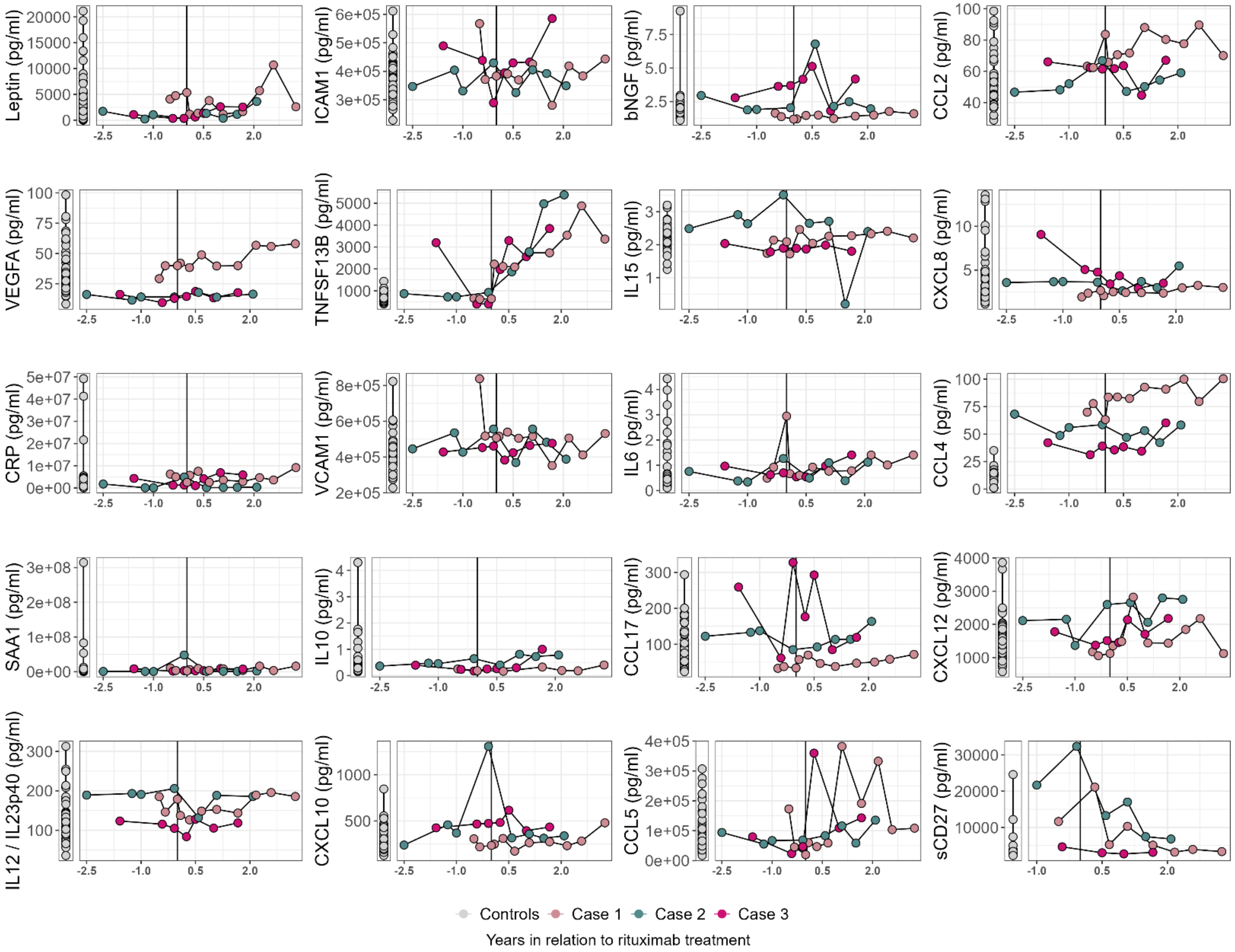

Supplement: Supplementary file 2 — Supplementary Figure 2 [file 41380_2024_2750_MOESM2_ESM.pdf]
