# Supplementary material for: Three cases with chronic obsessive compulsive disorder report gains in wellbeing and function following rituximab treatment
Source: Mol Psychiatry. 2024 Sep 21;30(4):1396–406. doi: 10.1038/s41380-024-02750-y (PMC11919689; doi:10.1038/s41380-024-02750-y)
Supplement: Supplementary file 3 — Legends for Supplementary figures [file 41380_2024_2750_MOESM3_ESM.docx]

**Legends for Supplementary figures**

**Supplementary Fig 1.** Analysis of immunological marker levels in CSF for cases before and after rituximab treatment compared to healthy controls (n=6). All time points before treatment and after treatment are represented.

**Supplementary Fig 2.** Analysis of immunological marker levels in plasma for cases before and after rituximab treatment compared to healthy controls (n=50). All time points before and after treatment are represented.
