# Supplementary material for: Three cases with chronic obsessive compulsive disorder report gains in wellbeing and function following rituximab treatment
Source: Mol Psychiatry. 2024 Sep 21;30(4):1396–406. doi: 10.1038/s41380-024-02750-y (PMC11919689; doi:10.1038/s41380-024-02750-y)
Supplement: Supplementary file 4 — Supplementary tables 1-3 [file 41380_2024_2750_MOESM4_ESM.docx]

**Supplementary tables**

| **Supplementary Table 1. Extended case data from before Rituximab treatment. OCD characteristics as assessed by Y­ BOCS affected functions as assessed by SDS and previous treatments.** | | | |
| --- | --- | --- | --- |
|  | **Case 1** | **Case 2** | **Case 3** |
| **Y-BOCS OBSESSIVE THOUGHTS** | 12p | 6p | 14p |
| **AGGRESSION** | - | - | Yes |
| **CONTAMINATION** | - | Yes | Yes |
| **SEXUALITY** | - | - | - |
| **HOARDING** | - | Yes | Yes |
| **RELIGION, MORALITY** | - | Yes | Yes |
| **SYMMETRY, EXACTNESS** | Yes | - | Yes |
| **BODY, DISEASE** | - | Yes | Yes |
| **Y-BOCS COMPULSIONS** | 12p | 13p | 12p |
| **CLEANING** | Yes | Yes | - |
| **CONTROLLING** | Yes | Yes | - |
| **REPEATING** | Yes | - | Yes |
| **COUNTING** | Yes | - | - |
| **ARRANGING** | Yes | - | - |
| **HOARDING** | - | - | - |
| **MENTAL OR PHYSICAL RITUALS** | - | Yes | Yes |
| **TRICHOTILLOMANINA** | - | - | - |
| **TICS** | Yes^1^ | Yes^2^ | - |
| **Affected functions (SDS)** | | | |
| **WORK/SCHOOL** | 8/10 | 10/10 | 10/10 |
| **SOCIAL LIFE/SPARE TIME** | 6/10 | 10/10 | 10/10 |
| **FAMILY LIFE, CHORES** | 5/10 | 10/10 | 10/10 |
| **Other** | | | |
| **"STUCK" IN ADL ACTIVITIES** | Yes | Yes | - |
| **PREOCCUPIED WITH HEALTH** | - | - | Yes |
| **Treatments before rituximab** | | | |
| **CBT** | Yes | Yes | Yes |
| **ANTIDEPRESSANTS/ANXIOLYTICS** | 3 (P, E, S) | 2 (C, Bup) | 2 (S, Bus) |
| **NEUROLEPTICS** | 1 (R) | 2 (A, Z) | 2 (A, Q) |
| **MOOD STABILIZERS** | No | 1 (T) | 3 (V, Lam, Lit) |
| **ANTIBIOTICS** | No | Frequent antibiotics for acute infections and prophylactic | Doxycycline |
| **IMMUNE MODULATORY** | No | IvIG (5 rounds) | No |
| **CNS STIMULANTS** | No | Yes (M, L, Am) | No |
| Abbreviations: Y-BOCS: Yale-Brown Obsessive-Compulsive Scale, SDS, Sheehan Disability Scale (10 points= maximum disability), ADL: Activities of Daily Life, CBT: Cognitive Behavioral Therapy  1 Knocking, touching  2 Knocking, touching, oral, sexual, vocal  Antidepressant and anxiolytics: P: Paroxetin; E: Escitalopram; C: Citalapram; Bus: Buspiran; Bup: Bupropion. Neuroleptics: A: Aripiprazol; Q: Quetiapin; R: Risperidon; Z: Ziprasidon.  Mood stabilizers: T.· Tapiramat; V: Valproat; Lam: Lamotrigin; Lit: Litium.  CNS-stimulants: M: Methylphenidate, L: Lisdexamfetamine, Am: Amantadine. /VIG: intravenous immune glabuline; PcV: Penicillin V | | | |

| **Supplementary Table 2. List of proteins measured in cerebrospinal fluid and plasma.** | | |
| --- | --- | --- |
| **Protein name** | **Coefficient of variation (CV)** | **Panel/Product no./Manufacturer** |
| C reactive protein (CRP) | 3% | Vascular Injury/ K15198D/Mesoscale |
| B-cell activating factor (BAFF) | 5% | U-plex/Mesoscale |
| Nerve growth factor (NGF) | 7% | U-plex/Mesoscale |
| lntercellular Adhesion Molecule 1 (ICAM) | 7% | Vascular Injury/ K15198D/Mesoscale |
| Interferon y (IFN-y) | 4% | Proinflammatory/K15049D/Mesoscale |
| Interleukin 10 (IL-10) | 4% | Proinflammatory/K15049D/Mesoscale |
| Interleukin IL-12/IL-23p40 (IL-12/IL-23p40) | 4% | Cytokine/K151A0H/Mesoscale |
| Interleukin 15 (IL-15) | 4% | Cytokine/K151A0H/Mesoscale |
| Interleukin 6 (IL-6) | 5% | Cytokine/K151A0H/Mesoscale |
| Interleukin 8 (IL-8) | 5% | Cytokine/K151A0H/Mesoscale |
| Leptin | 3% | U-plex/Mesoscale |
| Monocyte Chemotactic Protein-1 (MCP1) | 7% | Chemokine/K15047D/Mesoscale |
| Macrophage Inflammatory Protein 1 (MIP-1) | 7% | Chemokine/K15047D/Mesoscale |
| Regulated upon Activation, Normal Tcell Expressed and Secreted (RANTES) | 7% | U-plex/Mesoscale |
| Serum amyloid A (SAA) | 7% | Vascular Injury/ K15198D/Mesoscale |
| Thymus activation regulated chemokine (TARC) | 7% | Chemokine/K15047D/Mesoscale |
| Vascular cell adhesion protein (VCAM) | 6% | Vascular Injury/ K15198D/Mesoscale |
| Vascular endothelial growth factor (VEGF) | 3% | Cytokine/K151A0H/Mesoscale |
| CD27 | 5% | CD27/DuoSet Elisa DY382-05/RnD System |
| List of proteins with detectable values present in at least 80% of all cases in the cerebrospinal fluid (CSF) samples. Excluded proteins (less than 80% detectable values in CSF) are; Brain-derived neurotrophic factor (BDNF), Interleukin 17A (IL-17A), Interleukin 1 beta (IL-1), Interleukin 4 (IL-4), Macrophage Inflammatory Protein 1a (MIP-1a), Stromal cell-derived factor-1 (SDF-1a), Tumor necrosis factor alpha (TNF-a) and Granulocyte Macrophage Colony-Stimulating Factor (GM-CSF). Interferon a2a (IFN-a2a) was excluded due to technical issues. U-plex is a custom-made assay with analytes selected by the customer. All assays are bought from Mesoscale, except CD27 that is from RnD Systems, UK. | | |

**Supplementary Table 3a.** Pathways enriched via pathway over representation analysis (ORA) and gene set enrichment analysis (GSEA) and their statistical significance.

| WikiPathway ID | Term Name | q-value (GSEA) | q-value (ORA) | Significantly regulated genes |
| --- | --- | --- | --- | --- |
| WP558 | Complement and coagulation cascades | 1.6E-6 | 0.048 | SERPING1/C1R/C4B/C9/C8G/CFH |
| WP545 | Complement activation | 3.3E-6 | 0.122 | C1R/C4B/C9/C8G |
| WP5090 | Complement system in neuronal development and plasticity | 2.6E-5 | 0.048 | SERPING1/C1R/C4B/C9/C8G/CFH |
| WP2328 | Allograft rejection | 8.9E-5 |  | C4B/C9 |
| WP2806 | Complement system | 4.4E-4 |  | SERPING1/C9/CFH |
| WP4754 | IL-18 signaling pathway | 0.0061 |  | B2M |
| WP5115 | Network map of SARS-CoV-2 signaling pathway | 0.025 |  | CD163/ACTB/C1R/CFH |

**Supplementary Table 3b.** Pathways enriched via mapping the top 20 differentially expressed proteins onto pathways using multiple bioinformatics solutions: Qiagen IPA, G-profiler, String database and Oracle healthcare data model. All pathway solutions found that the most differentially expressed biological process is the complement pathway.

| ID Source | Term ID | Term name | P (adj) |
| --- | --- | --- | --- |
| GO:MF | GO:0001848 | Complement binding | 5.0E-7 |
| GO:MF | GO:0097001 | Ceramide binding | 2.4E-3 |
| GO:MF | GO:0001846 | Opsonin binding | 3.3E-3 |
| GO:BP | GO:0006956 | Complement activation | 2.4E-7 |
| GO:BP | GO:0045807 | Positive regulation of endocytosis | 1.9E-2 |
| GO:CC | GO:0005576 | Extracellular region | 1.9E-15 |
| GO:CC | GO:0044217 | Other organism part | 1.2E-6 |
| GO:CC | GO:0045202 | Synapse | 1.5E-3 |
| GO:CC | GO:0030424 | Axon | 4.1E-3 |
| GO:CC | GO:0005579 | Membrane attack complex | 1.5E-2 |
| GO:CC | GO:0005793 | Endoplasmic reticulum-Golgi intermediate compartment | 2.4E-2 |
| GO: Gene Ontology; MF: Molecular function; BP: biological processes; CC: cellular components for these proteins as derived from the G profiler database. | | | |
